# The Role of Regulatory T Cells in Periodontitis With or Without Systemic Diseases: A Systematic Review

**DOI:** 10.3390/ijms27188415

**Published:** 2026-09-21

**Authors:** Angelo Michele Inchingolo, Maria Celeste Fatone, Grazia Marinelli, Laura Ferrante, Francesca Calò, Andrea Palermo, Marco Severino, Francesco Inchingolo, Alessio Danilo Inchingolo, Gianna Dipalma

**Affiliations:** 1 Department of Medicine and Surgery, University of Perugia, 06132 Perugia, Italy; 2 Presidio Territoriale di AssistenzaTrani-ASL BT, Viale Padre Pio, 76125 Trani, Italy; maria.fatone@aslbat.it; 3 Department of Interdisciplinary Medicine, University of Bari “Aldo Moro”, 70124 Bari, Italy; graziamarinelli@live.it (G.M.); lauraferrante79@virgilio.it (L.F.); francesca.calo@uniba.it (F.C.); francesco.inchingolo@uniba.it (F.I.);; 4 Department of Experimental Medicine, University of Salento, 73100 Lecce, Italy; andrea.palermo@unisalento.it; 5 Department of Life Science, Health and Health Professional, Link Campus University, 00165 Rome, Italy; g.dipalma@unilink.it

**Keywords:** cytokines, periodontitis, regulatory T cells, systemic diseases, systemic inflammation, Th17

## Abstract

Periodontitis is a chronic immune-mediated inflammatory disease of the oral cavity, frequently linked to systemic conditions through complex immunological pathways. Regulatory T lymphocytes (Tregs) are pivotal in maintaining immune homeostasis by producing anti-inflammatory mediators and enforcing self-tolerance. Recent research has highlighted their role in inflammatory, immune-mediated, and neoplastic diseases. This systematic review followed the Preferred Reporting Items for Systematic Reviews and Meta-Analyses (PRISMA) guidelines, exploring the role of Tregs in periodontitis, including patients with comorbidities such as type 2 diabetes mellitus, rheumatoid arthritis, atherosclerosis, and cancer, with a focus on cytokine profile alterations and therapeutic implications of Treg modulation. A systematic search of PubMed, Scopus, Web of Science, and Cochrane from January 2015 to August 2025 using “periodontitis” and “Tregs” or “regulatory T cells” yielded 20 studies. Available evidence suggests alterations in the balance between immunomodulatory Tregs and pro-inflammatory Th17 lymphocytes, together with changes in related cytokine profiles compared with healthy controls. Changes in Treg-related immune markers have also been reported following periodontal interventions; however, these findings do not establish a causal role for Treg modulation or support Treg-targeted therapies in current clinical practice. Well-designed prospective studies and, where appropriate, randomized controlled trials are required to strengthen causal and therapeutic inference.

## 1. Introduction

Periodontitis is a chronic, immune-mediated inflammatory disease affecting the supporting tissues of the teeth and is characterized by gingival inflammation, periodontal pocket formation, progressive attachment loss, and alveolar bone resorption [1]. Its pathogenesis results from a dysregulated host response to a dysbiotic subgingival microbiota [2,3,4,5]. Under physiological conditions, the periodontal immune environment maintains a dynamic balance between effector and regulatory mechanisms that permits microbial surveillance while limiting excessive tissue-damaging inflammation [6,7,8]. In periodontitis, disruption of this balance promotes persistent inflammatory responses and progressive periodontal destruction [9,10,11,12,13].

Among the adaptive immune pathways involved, the balance between T helper 17 (Th17) cells and regulatory T cells (Tregs) has received increasing attention [14,15,16,17]. Th17-related responses are associated with pro-inflammatory signaling and tissue inflammation, whereas Tregs contribute to immune regulation and maintenance of tolerance [18,19,20,21,22,23,24,25,26]. Experimental and clinical evidence indicates that alterations in the frequency, phenotype, or stability of these populations may occur in periodontitis, although the direction and biological significance of these changes vary according to the clinical context, biological compartment, and analytical method [27,28,29,30,31,32]. These alterations may also be relevant to the systemic inflammatory burden associated with periodontitis and with comorbid conditions such as diabetes mellitus, rheumatoid arthritis, cardiovascular disease, and cancer [33,34,35,36].

Tregs are a specialized subset of CD4^+^ T lymphocytes with a central role in maintaining immune homeostasis and self-tolerance [37,38,39,40,41,42,43]. The term Cluster of Differentiation (CD) is used throughout the manuscript for cell-surface markers [44,45,46,47,48]. In human studies, Tregs are commonly characterized by high CD25 expression together with FOXP3 and, where assessed, low CD127 expression, whereas murine studies frequently identify them as CD4^+^CD25^+^FOXP3^+^ cells [49,50,51,52,53]. Importantly, CD25 is not specific to Tregs, because it may also be transiently expressed by activated conventional effector T cells; therefore, CD25 expression alone should not be considered sufficient to define a regulatory T-cell population [54,55,56,57,58]. This distinction is particularly relevant when comparing studies employing different immunophenotyping strategies [59].

Tregs exert suppressive activity through several complementary mechanisms, including secretion of regulatory cytokines such as interleukin-10 (IL-10), transforming growth factor beta (TGF-β), and interleukin-35 (IL-35); high-affinity consumption of IL-2 through CD25; CTLA-4-mediated modulation of antigen-presenting cells; and metabolic or adenosine-dependent suppression of effector-cell responses [60,61,62,63]. Primary references for the individual mechanisms will be checked and positioned directly after the corresponding statements during the final reference revision [64,65,66,67,68,69,70]. FOXP3 is the lineage-defining transcription factor most closely associated with Treg development and suppressive function. Its biological relevance is illustrated by Immune dysregulation, Polyendocrinopathy, Enteropathy, X-linked (IPEX) syndrome, in which loss-of-function FOXP3 mutations result in severe early-onset autoimmunity, typically including enteropathy, eczema, and autoimmune endocrinopathy [71,72,73,74,75].

The biological role of Tregs has also prompted investigation of strategies aimed at enhancing their number, stability, or regulatory activity [76,77,78,79,80]. However, evidence relevant specifically to periodontitis remains predominantly preclinical or exploratory [59,81]. Murine studies suggest that Treg expansion or adoptive transfer may attenuate periodontal inflammation and alveolar bone loss, but these observations cannot yet be directly extrapolated to routine periodontal care [82,83,84,85,86,87,88,89]. Potential pharmacological, microbiota-based, and cell-based approaches and their clinical feasibility are therefore considered critically in the Discussion rather than regarded as established Treg-targeted periodontal therapies [90,91,92,93].

This systematic review aims to provide a focused synthesis of contemporary evidence on the role of Tregs in the onset and maintenance of periodontitis, both in isolation and in association with systemic diseases, with particular attention to their relationship with Th17-related responses and Treg-associated immunological endpoints [94,95,96,97,98]. A further objective is to evaluate changes in Treg-related immune markers following periodontal interventions and to critically assess the current evidence supporting the potential therapeutic relevance of Treg modulation.

## 2. Materials and Methods

### 2.1. PECO and PICO Questions

Given the heterogeneity of the included evidence, two complementary frameworks were used according to the nature of the research question.

For the etiological and mechanistic component of the review, a PECO framework was adopted:

- **Population (P):** adult patients;
- **Exposure (E):** marginal periodontitis, with or without associated systemic diseases;
- **Comparator (C):** periodontally healthy individuals and/or appropriate control groups without marginal periodontitis, depending on the study design;
- **Outcome (O):** alterations in Treg-related immune parameters, including Treg abundance and phenotype, Th17/Treg balance, Treg-associated cytokines, and related immunological markers.

The corresponding PECO question was: In adults, is marginal periodontitis, with or without associated systemic diseases, associated with alterations in Treg-related immune parameters compared with periodontally healthy or otherwise appropriate control groups?

For the therapeutic component of the review, a PICO framework was used:

- **Population (P):** adult patients with marginal periodontitis;
- **Intervention (I):** periodontal interventions evaluated in the included studies;
- **Comparator (C):** pretreatment baseline and/or patients with periodontitis not receiving the adjunctive intervention, as applicable to the individual study design;
- **Outcome (O):** changes in Treg-related immune markers, Th17/Treg balance, Treg-associated cytokines, and clinical periodontal outcomes.

The corresponding PICO question was: In adults with marginal periodontitis, what changes in Treg-related immune markers and clinical periodontal outcomes are observed following periodontal interventions compared with pretreatment baseline or an appropriate periodontitis control group?

### 2.2. Protocol and Registration

The review was conducted in accordance with the Preferred Reporting Items for Systematic Reviews and Meta-Analyses (PRISMA) guidelines and was registered in the International Prospective Register of Systematic Reviews (PROSPERO) (ID: 1150470).

### 2.3. Search Strategy

A systematic literature search was conducted in PubMed/MEDLINE, Scopus, Web of Science Core Collection, and the Cochrane Library to identify studies addressing regulatory T cells in marginal periodontitis. The search was based on two main concepts: periodontitis (“periodontitis” and “periodontal disease”) and regulatory T cells (“regulatory T cells”, “regulatory T lymphocytes”, “Treg”, and “Tregs”). The two concepts were combined using the Boolean operator AND, while synonymous terms within each concept were combined using OR. The search approach was adapted to the characteristics of each database. Searches were restricted to English-language studies published between 1 January 2015 and 22 August 2025. The databases searched, search concepts, applied limits, and number of records retrieved are summarized in Table 1.

### 2.4. Inclusion and Exclusion Criteria

The inclusion criteria were the following: (1) human subjects; (2) English language; (3) randomized controlled trials (RCTs), clinical trials, cohort studies, and observational studies; (4) adult patients affected by marginal periodontitis with or without systemic diseases; (5) studies evaluating periodontal interventions and reporting changes in Treg-related immune markers and/or associated clinical periodontal outcomes; (6) timespan from 2015 to 2025. The publication period from 2015 to 2025 was predefined to provide a focused synthesis of contemporary evidence on Treg-related immune alterations in periodontitis. This time frame was selected to capture studies employing more recent immunophenotyping, molecular, and analytical approaches and to reflect current concepts regarding the role of Tregs in periodontal and systemic inflammatory conditions.

In parallel, the exclusion criteria were the following: (1) animal models; (2) other languages except English; (3) qualitative studies, reviews, meta-analyses, case reports, case series, editorials, and consensus reports; (4) studies exclusively addressing gingivitis, apical periodontitis or periapical lesions, peri-implant diseases, or other oral inflammatory conditions without a marginal periodontitis group; (5) in vitro studies; (6) articles prior to 2015.

The restriction to English-language publications was applied to ensure consistent and reliable full-text assessment by the review team.

Throughout this review, the term “periodontitis” refers to marginal periodontitis. Gingivitis, apical periodontitis, periapical lesions, and peri-implant diseases were considered distinct clinical entities and were not treated as interchangeable with marginal periodontitis.

### 2.5. Data Processing

Two reviewers (M.C.F. and F.C.) subsequently examined the articles applying the inclusion and exclusion criteria. Any criticism was cleared up through the consultation of two senior reviewers (F.I. and G.D.). The selected articles were schematized in an Excel table and downloaded into reference manager software, in this case, Zotero (version 6.0.15).

### 2.6. Risk of Bias Assessment

Risk of bias was assessed using study-design-specific instruments. Analytical cross-sectional studies were evaluated using the Joanna Briggs Institute (JBI) critical appraisal tool for analytical cross-sectional studies, whereas cohort and retrospective cohort studies were assessed using the corresponding JBI critical appraisal tool for cohort studies. Non-randomized interventional studies were assessed using the Risk Of Bias In Non-randomized Studies of Interventions (ROBINS-I) tool. Randomized controlled trials were assessed using the Cochrane Risk of Bias 2 (RoB 2) tool. The two-sample Mendelian randomization study was appraised separately using an MR-specific domain-based framework, with particular consideration of instrument relevance, independence, horizontal pleiotropy, population structure and sample overlap, and the robustness of sensitivity analyses.

**Figure 1:**
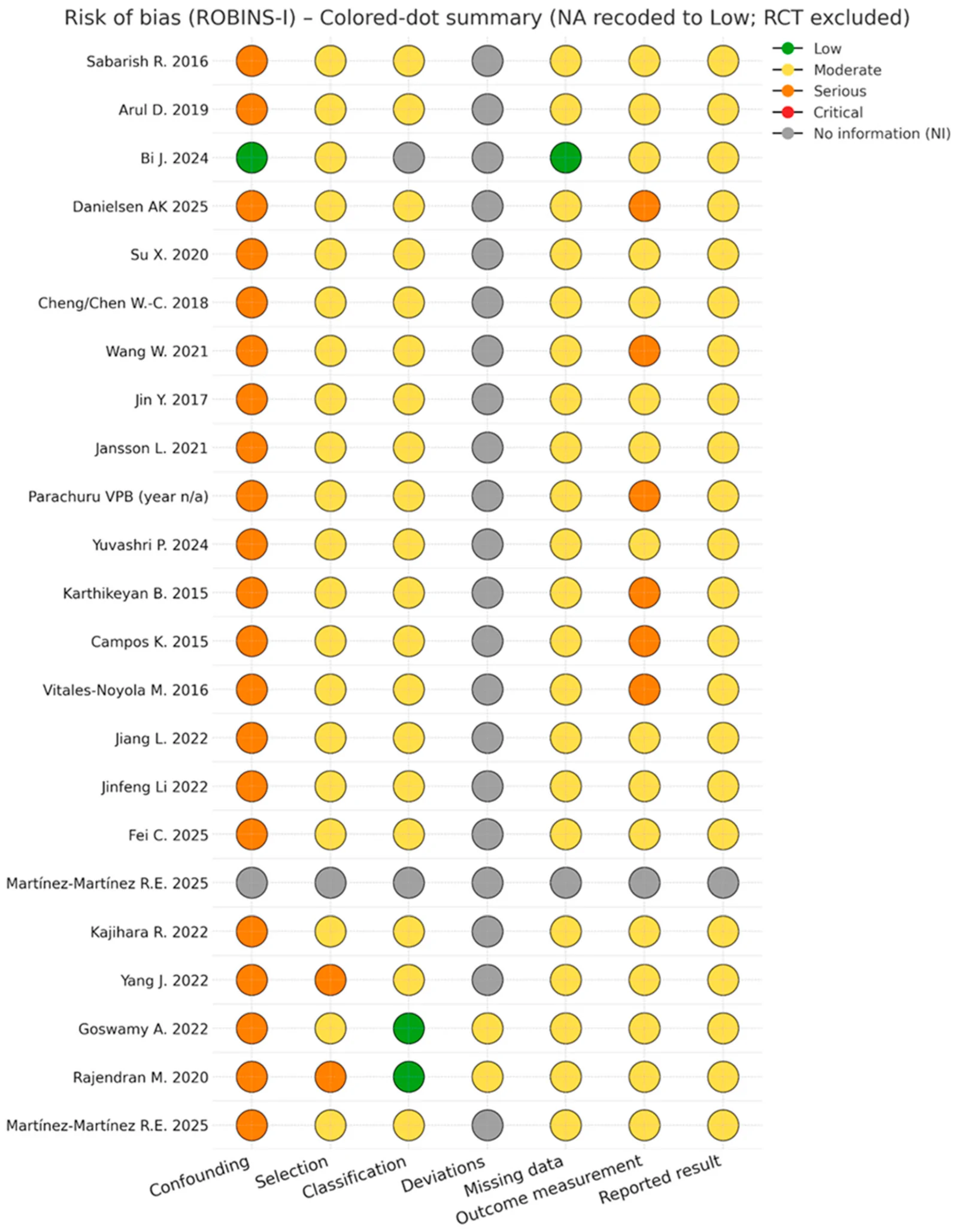

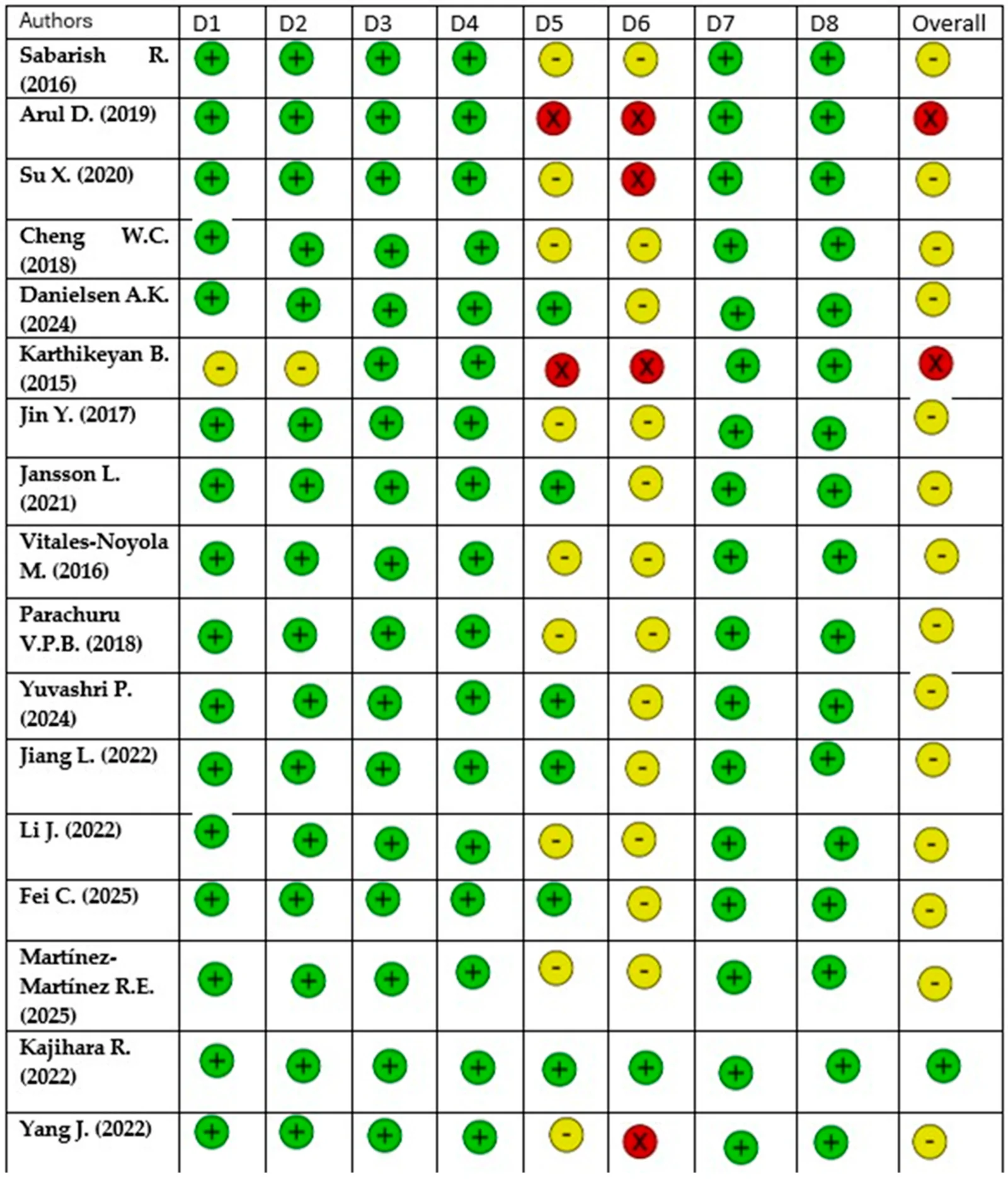

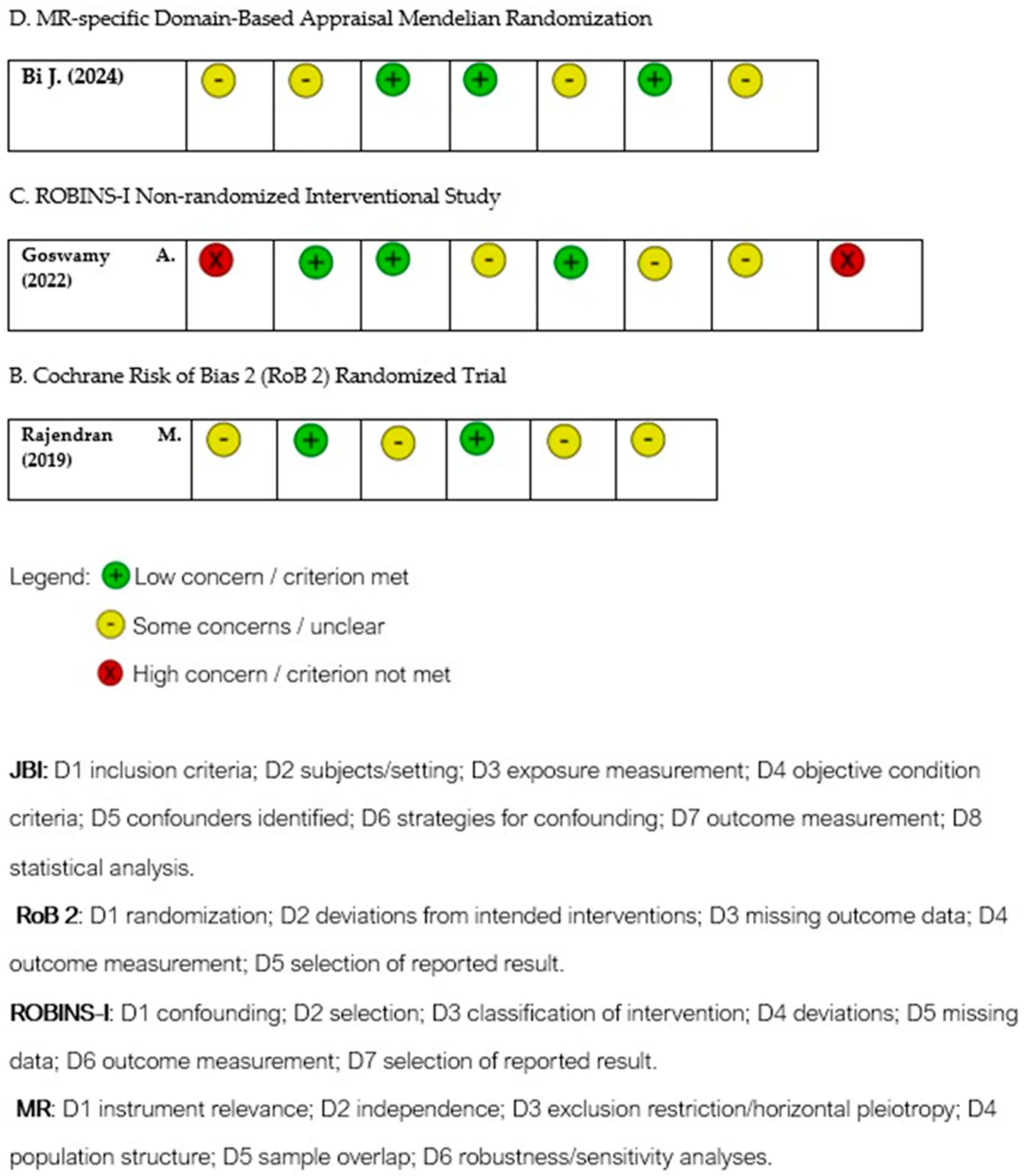
Risk-of-bias assessment of the included studies using study-design-specific appraisal tools [99,100,101,102,103,104,105,106,107,108,109,110,111,112,113,114,115,116,117,118,119,120].

Risk-of-bias assessments were performed independently by two reviewers (F.I. and G.D.). Any disagreements were resolved through discussion and consensus. Domain-level risk-of-bias judgements are presented in Figure 1.

### 2.7. Evidence Synthesis

Given the substantial clinical and methodological heterogeneity among the included studies, the findings were synthesized narratively rather than quantitatively. Studies were first grouped according to the main clinical domain addressed: (1) Treg-related alterations in marginal periodontitis without specific systemic comorbidities; (2) Treg-related alterations in marginal periodontitis associated with systemic diseases; and (3) changes in Treg-related immune markers following periodontal interventions.

Within these domains, findings were further interpreted according to study design, biological compartment and specimen type (e.g., peripheral blood, gingival tissue, gingival crevicular fluid, or saliva), the definition and measurement of Treg-related endpoints, periodontal phenotype, systemic condition, and, where applicable, type of intervention and follow-up duration. Treg-related outcomes were considered according to the specific biological parameter assessed, including cellular abundance or phenotype, Th17/Treg balance, Treg-associated cytokines, and other related immunological markers.

A meta-analysis was not performed because of substantial heterogeneity across studies in population characteristics, periodontal and systemic disease definitions, study designs, biological specimens, Treg phenotyping and analytical methods, reported outcomes, interventions, and follow-up periods. These differences precluded the identification of sufficiently homogeneous groups of studies for a clinically meaningful quantitative pooling of effect estimates.

## 3. Results

### 3.1. Search Results

The database searches yielded a total of 364 records across PubMed/MEDLINE, Scopus, Web of Science Core Collection, and the Cochrane Library. Following removal of 204 duplicate records, 160 unique records remained and underwent title and abstract screening. The screening phase consisted of the evaluation of the title and abstract based on inclusion and exclusion criteria. Studies focusing on Treg-related immune profiles in marginal periodontitis, with or without associated systemic diseases, as well as on changes in Treg-related markers following periodontal interventions, were considered potentially eligible.

Studies exclusively addressing oral inflammatory conditions other than marginal periodontitis, such as gingivitis, apical/periapical disease, or stomatitis, without an eligible marginal periodontitis group were considered outside the predefined disease scope and were excluded.

**Figure 2:**
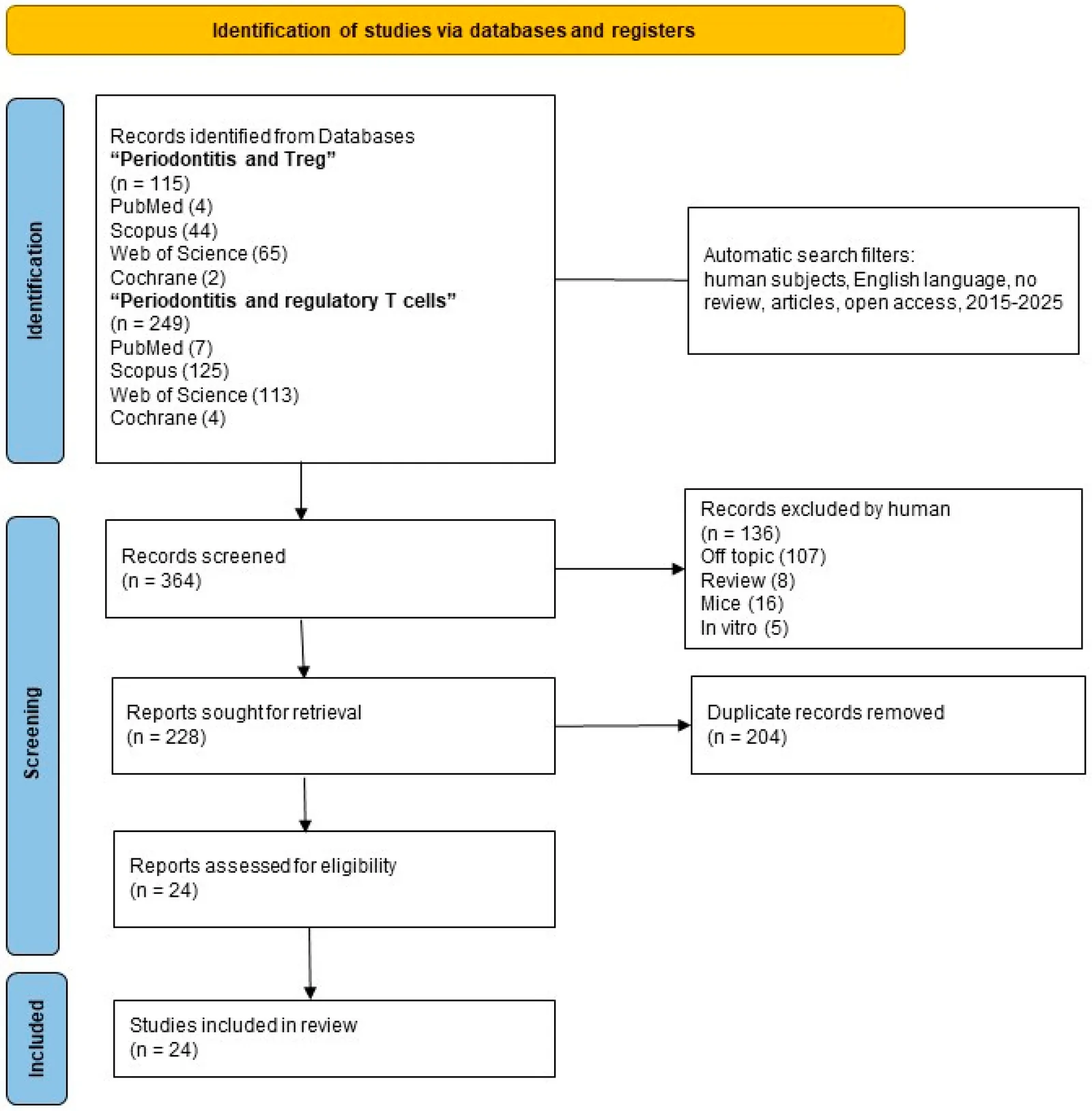
Preferred Reporting Items for Systematic Reviews and Meta-Analyses (PRISMA) 2020 flow-chart.

Of the 160 records screened, 136 were excluded because they did not meet the predefined eligibility criteria. The full texts of 24 reports were subsequently retrieved and assessed for eligibility. Following full-text assessment, four reports were excluded, resulting in 20 studies being included in the final qualitative synthesis (Figure 2).

### 3.2. Risk of Bias Results

Risk of bias was assessed using study-design-specific instruments, as described in Section 2.6. Among the analytical cross-sectional studies, most fulfilled the criteria related to clearly defined inclusion criteria, appropriate measurement of exposures and outcomes, and adequate statistical analysis. The main methodological concerns involved the identification and control of potential confounding factors and, in some studies, incomplete reporting of strategies used to address confounding. The randomized pilot trial was assessed using the Cochrane RoB 2 tool and showed some concerns across selected domains. The non-randomized interventional study showed a higher risk of bias, mainly because of the absence of a concurrent control group and the potential influence of confounding factors. The Mendelian randomization study was appraised separately using an MR-specific framework and showed some concerns related to the assumptions underlying instrumental-variable analyses, although sensitivity analyses supported the robustness of the reported findings. Overall, the methodological quality of the included evidence was heterogeneous, and the risk-of-bias assessment supports a cautious interpretation of causal and therapeutic inferences. Detailed domain-level judgements are presented in Figure 1.

## 4. Discussion

### 4.1. Alteration of Regulatory T Lymphocytes (Treg) and Treg-Related Cytokine Profile in Periodontitis Patients

Periodontitis is characterized by an altered host immune response that plays a crucial pathogenic role in the destruction of periodontal tissues [121,122,123,124,125,126,127,128]. At the core of this dysfunctional process is a delicate balance between pro-inflammatory and immunoregulatory T-cell subpopulations [129,130,131,132,133,134,135,136]. Recent studies have shifted the focus to the critical role of the T helper 17 (Th17) to regulatory T-cell (Treg) ratio, which serves as promoters and suppressors of inflammation, respectively. Th17 cells, characterized by the expression of the transcription factor retinoic acid-related orphan receptor gamma t (RORγt) and the production of pro-inflammatory cytokines such as IL-17A, have been directly correlated with the pathogenesis of periodontitis and alveolar bone destruction [137,138,139,140,141,142]. Conversely, Treg cells, characterized by the FoxP3 transcription factor and the release of anti-inflammatory cytokines like IL-10 and TGF-β, are essential for maintaining immunological homeostasis and suppressing the inflammatory response. An alteration of this balance, leading to a dominance of Th17 responses, is considered a key factor in disease progression [143,144,145,146,147]. Therefore, although this review focuses on the role of Treg lymphocytes in periodontitis, it is essential to discuss the role of Th17 lymphocytes in relation to them [148,149,150,151,152,153,154,155,156].

Importantly, the Treg-related endpoints evaluated across the included studies represent distinct biological dimensions and should not be interpreted as interchangeable measures of Treg activity. Circulating or tissue Treg frequency reflects cellular abundance, whereas the expression of phenotypic markers such as CD25, FOXP3, or CD69 provides information on cellular phenotype but does not necessarily demonstrate suppressive function. Similarly, the Th17/Treg ratio describes the relative distribution of two immune-cell populations, while FOXP3 gene expression reflects transcriptional regulation. Treg plasticity refers to phenotypic and functional instability, including the acquisition of Th17-like features. Finally, cytokines such as IL-10, TGF-β, and IL-35 are associated with regulatory immune responses but are not exclusively produced by Tregs and therefore cannot be considered direct surrogate measures of Treg abundance or suppressive activity. These distinctions were considered when interpreting apparently divergent findings across studies.

An additional source of methodological heterogeneity arises from the different flow-cytometry gating strategies used to identify Tregs across studies. Populations defined as CD4^+^CD25^+^, CD4^+^FOXP3^+^, or CD4^+^CD25^+^FOXP3^+^ should not be considered biologically equivalent. In particular, CD25 may also be transiently expressed by activated conventional T cells, whereas FOXP3 expression alone may not always correspond to a stable suppressive phenotype. More restrictive multiparametric phenotyping can therefore provide greater specificity for Treg identification. Differences in gating strategies may have contributed to variability in the reported frequency of Tregs and should be considered when comparing findings across studies. As regards the role and the levels of Tregs and their cytokines in periodontitis, the data are not always consistent. In a pilot study, Sabarish R et al. were among the first to observe that serum levels of CD4CD25 foxp3-expressing regulatory T cells are significantly higher in subjects with chronic periodontitis compared to healthy controls, using flow cytometry [99]. Similarly, Arul D. et al. confirmed that the levels of CD4^+^, CD25^+^ e Fox P3^+^ Treg cells were significantly higher in the peripheral blood of periodontitis patients compared to patients with gingivitis and to healthy controls [100]. The recent study by Bi J et al. was the first to measure the causal relationship between immune cells and periodontitis using two-sample Mendelian randomization as the analysis method. Specifically, the data confirmed the association between the onset and progression of periodontitis and most types of Treg cells (the OR of the risk of CD25 over CD39^+^CD4^+^ T cells was 1.058) [101].

Some authors observed a reduction in the subpopulation of IL-10-producing T cells (Treg) in grade B and C periodontitis patients compared to healthy controls, before and after stimulation with *P. gingivalis*, *F. nucleatum* or the commensal bacteria, Staphylococcus epidermidis and Cutibacterium acnes. In parallel, an elevated number of interferon-gamma (IFN-γ) producing T cells (Th17) was observed before and after stimulation and was correlated with clinical attachment loss (CAL) [102]. According to Su X et al., the proportion of Treg cells was significantly reduced in periodontitis patients compared to healthy controls, whereas the proportion of Th17 cells and TH17/Treg ratio were significantly higher in periodontitis patients than in healthy controls. Specifically, younger periodontitis patients (aged 18 to 40 years) presented lower Treg cell levels than older periodontitis patients (aged 40 to 76 years). Furthermore, in young people, the overexpression of CD40 on dendritic cells was correlated with Th17/Treg imbalance in chronic periodontitis. Similarly, the proportion of Th17 cells and the Th17/Treg ratio were significantly higher in the young group than in the elderly group. In confirmation of these findings, serum levels of CD40L and IL-17 were increased in patients with chronic periodontitis, especially in the younger patients [103].

Conversely, Chen WC et al. did not find significant differences in the proportion of CD4^+^CD45RO^+^CD25^+^CD127Tregs in the peripheral blood of periodontitis patients compared to healthy controls, as well as in CD4^+^ T cells, CD8^+^ T cells, γδ T cells, CD19^+^ B cells, CD14^+^ monocytes, cytokine-producing T cells, and in the phenotype of CD14^+^ monocytes [104].

Local and compartment-specific Treg-associated findings were also evaluated in gingival crevicular fluid, gingival tissue, and saliva, although some studies simultaneously assessed systemic blood-derived markers. Jin et al. reported higher IL-35 levels in both systemic and local compartments, with increased concentrations in serum and gingival crevicular fluid (GCF), respectively. The study also evaluated IL-35-related molecular expression in blood- and periodontal tissue-derived samples. These findings should be interpreted according to the biological compartment examined rather than as equivalent measures of Treg activity [105]. Jansson L et al. studied the intra-individual cytokine profile of GCF in subjects with periodontitis and peri-implantitis. Regarding immunomodulatory cytokines produced by Treg cells, namely IL-35, IL-11 and IL-10, their levels were frequently lower in healthy dental and implant sites than in sites with periodontitis or peri-implantitis [106]. According to Parachuru VPB et al., Treg cells show a more important role than Th17 cells in the pathogenesis of periodontitis. The authors found that in inflamed periodontal tissues, cells expressing Foxp3 predominate over cells producing IL-17. They also found higher levels of TGFβ1 and IL-10 in periodontal tissues and gingival tissues with more B cells and plasma cells compared to healthy/gingivitis tissues and gingival tissues where T cells predominate. IL17^+^ cells belong to the mast cell class rather than Th17 cells, reducing the role of the latter in the chronic inflammation underlying periodontitis [107].

The cross-sectional study by Yuvashri et al. analyzed salivary levels of IL-35, a cytokine associated with regulatory immune responses, in smokers and non-smokers with periodontitis. IL-35 was significantly higher in periodontitis patients, particularly among non-smokers, and correlated with clinical parameters. However, smokers exhibited reduced IL-35 levels, suggesting tobacco-induced impairment of immunoregulation [108].

These findings suggest that IL-35 levels may reflect changes in the local regulatory immune environment and may be influenced by host and environmental factors such as smoking; however, salivary IL-35 cannot be considered a direct measure of Treg abundance or function.

Some authors have focused on the gene expression of Tregs. Naïve T helper (Th) cells differentiate into Th17 cells and Tregs based on the activation of RORγt and Foxp3, respectively. Karthikeyan B. quantified the gene expression of RORγt and Foxp3 in gingival tissues from periodontitis patients and healthy controls by reverse transcription polymerase chain reaction, revealing that Foxp3 is reduced by more than 6-fold in the periodontitis group [109]. However, changes in IL-10, TGF-β, or IL-35 concentrations should be interpreted as alterations in the regulatory cytokine milieu rather than as direct evidence of changes in Treg number or suppressive function.

In addition to conventional CD4^+^CD25^+^ Tregs, other regulatory T-cell phenotypes expressing FOXP3 and CD69^+^ have also been investigated in periodontitis. Vitales-Noyola et al. reported significantly higher frequencies of CD69^+^ Treg cells in both peripheral blood and gingival tissue of patients with periodontitis compared with healthy controls. Furthermore, these frequencies were inversely associated with periodontal attachment loss [110] (Table 2).

Taken together, the available studies do not support a single uniform directional change in Treg-related responses in periodontitis. Depending on the study, Treg frequency or Treg-associated markers were increased, decreased, or unchanged, while alterations in the Th17/Treg ratio were not necessarily accompanied by corresponding changes in absolute Treg abundance. Several factors may account for these apparently conflicting findings. First, Tregs were assessed in different biological compartments, including peripheral blood, gingival tissue, gingival crevicular fluid, and saliva; local recruitment of regulatory cells into inflamed periodontal tissues may therefore coexist with unchanged or different systemic profiles. Second, an increased number of phenotypically defined Tregs does not necessarily imply preserved suppressive function, because recruitment or expansion may represent a compensatory response to inflammation while functional impairment or phenotypic instability persists. Third, differences in periodontal severity, age, smoking status, systemic comorbidities, previous or ongoing treatment, and other patient-related factors may influence the observed immune profile. Finally, considerable methodological heterogeneity exists in the definition of Tregs and in the immunophenotyping and analytical approaches used across studies. Therefore, differences in Treg abundance, phenotype, transcriptional markers, cytokine profiles, and Th17/Treg balance should be interpreted within their specific biological and methodological context rather than as manifestations of a single uniform immunological alteration.

### 4.2. Alteration of Regulatory T Lymphocytes (Treg) in Periodontitis Patients with Systemic Diseases

The selected studies collectively underscore the multifaceted nature of the periodontitis–systemic disease axis, revealing distinct and converging immunoinflammatory mechanisms. Despite their diverse clinical contexts—type 2 diabetes mellitus (T2DM), rheumatoid arthritis (RA), cancer, and atherosclerosis—all studies converge on the mechanism of disruption of immune homeostasis, involving the Th17/Treg balance, cytokine signaling, and host–microbiome interactions.

The original research by Jiang et al. demonstrated that T2DM profoundly modifies the periodontal immune–microbial interface through a multi-omics approach integrating 16S rRNA sequencing, Ultra High-Performance Liquid Chromatography–Mass Spectrometry (UHPLC–MS) metabolomics, and flow cytometry. Specifically, T2DM patients with periodontitis showed a reduced Treg/Th17 ratio and increased Programmed Cell Death Protein 1/Programmed Death-Ligand 1 (PD-1/PD-L1) expression in CD4^+^ and CD8^+^ T cells, suggesting a chronic immunosuppressive state of inflammation. The metabolic signatures (involving butyrate, phenylalanine, and tryptophan metabolism) further supported the idea of a metabolic–immune dysregulation linking glycemic imbalance, dysbiotic microbiota, and host immune dysfunction [111].

The findings of Jinfeng Li et al. showed that T2DM was associated with alterations in Th1 and Th17/Treg-related immune profiles in the presence of periodontitis. The authors observed significant upregulation of Th1/Th2 and Th17/Treg ratios, particularly in patients with both conditions, consistent with a potentially enhanced pro-inflammatory immune profile in patients presenting both conditions. This reinforces the notion that T2DM predisposes individuals to exaggerated periodontal inflammation through systemic immune priming [112]. These studies consolidate the concept that metabolic dysregulation and periodontal infection cooperatively shape immune polarization, supporting integrated therapeutic approaches addressing both metabolic and periodontal control.

The recent cross-sectional cohort study by Fei et al. reported altered serum and salivary inflammatory and immunoregulatory mediator profiles in patients with rheumatoid arthritis and severe periodontitis, including increased levels of sCD30, IL-10, IL-19, and osteopontin, together with increased salivary BAFF/TNFSF13B and IFN-α2. These cytokines and tumor necrosis factor (TNF) superfamily members mark B-cell hyperactivation and regulatory feedback responses, pointing to a mutual amplification loop between periodontal and systemic inflammation. The strong association of osteopontin with bone resorption and BAFF/APRIL with autoimmunity supports the assumption that periodontitis acts as an additional inflammatory burden in RA, exacerbating immune and tissue damage [113].

In contrast, the recent clinical study by Martínez-Martínez et al. revealed that patients with RA and periodontitis had significantly higher Th17 cell percentages and greater *P. gingivalis* load, without a compensatory rise in Tregs. This imbalance, linked to *P. gingivalis*-mediated citrullination via the protein arginine deiminase (PAD) enzyme, provides a plausible mechanism connecting periodontal infection to autoantibody formation and Th17-driven inflammation in RA. These findings support a potential mechanistic link between *P. gingivalis* burden, altered Th17/Treg balance, and systemic autoimmune inflammation in patients with RA and periodontitis [114].

The retrospective study by Yang et al. addressed the relationship between periodontitis and atherosclerosis, a chronic and progressive inflammatory vascular disease. *P. gingivalis* infection was associated with reduced IDO activity and an altered Th17/Treg-related profile. Patients with atherosclerosis and *P. gingivalis* infection showed increased Th17 (RORγt, IL-17A) expression and decreased Treg (FoxP3, IL-10, TGF-β1) markers, as well as reduced IDO activity. The decline in IDO activity correlated negatively with the Th17/Treg ratio, implicating tryptophan–kynurenine metabolism in systemic immune dysregulation. These findings support a potential mechanistic association between periodontal infection, altered immune regulation, and cardiovascular inflammation [115].

The cross-sectional study by Kajihara et al. extended the systemic implications of periodontal inflammation to oncology, showing that patients with both cancer and periodontitis exhibited markedly elevated IL-6 and expanded Treg populations compared to other groups. These results indicate that the coexistence of periodontitis and cancer was associated with increased IL-6 levels and greater circulating Treg frequency. The absence of significant changes in vascular endothelial growth factor (VEGF), transforming growth factor-beta1 (TGF-β1), and C-C Motif Chemokine Ligand 22 (CCL22) suggests that IL-6 and Tregs are the principal mediators of the oral–tumor immunological axis [116] (Table 3).

The heterogeneity observed in patients with systemic diseases further emphasizes the context-dependent nature of Treg-related responses. The presence and severity of systemic inflammation, glycemic control in diabetes, autoimmune activity and systemic therapies in rheumatoid arthritis, cardiovascular inflammatory burden, and cancer-related immune modulation may independently affect Treg frequency, phenotype, and function. Consequently, differences observed between patients with periodontitis alone and those with systemic comorbidities cannot be attributed exclusively to periodontal inflammation. Moreover, differences in biological compartment and immunophenotyping strategies across studies further limit direct comparisons. These findings should therefore be interpreted primarily as associations within distinct clinical contexts rather than as evidence of a uniform systemic Treg response to periodontitis.

### 4.3. Changes in Treg-Related Immune Markers Following Periodontal Interventions

The available interventional evidence suggests that conventional periodontal treatments may be accompanied by changes in Treg-related immune markers. However, these interventions were not specifically designed to target Tregs, and the observed immunological changes do not establish that Treg modulation mediated the clinical response to treatment.

The interventional clinical study by Goswamy et al. focused on the immunomodulatory role of IL-35 in periodontal healing following non-surgical periodontal treatment (NSPT), consisting of scaling and root planing (SRP). The authors demonstrated that NSPT led to an increase in IL-35 levels in GCF, as well as to clinical improvements in plaque index (PI), gingival index (GI), probing pocket depth (PPD), and CAL. The negative correlation between IL-35 levels and clinical periodontal parameters suggests that IL-35 may reflect treatment-associated changes in the local regulatory immune environment. However, these findings do not demonstrate that IL-35 or Treg modulation directly mediated periodontal healing, nor are they sufficient to establish IL-35 as a validated biomarker of treatment response [117].

Finally, the pilot study by Rajendran et al. explored how short-term antibiotic treatment (AB) influences Treg/Th17 plasticity in periodontitis patients. The administration of metronidazole 250 mg plus amoxicillin 500 mg per os for 7 days in periodontitis patients reduced the conversion of Tregs into Th17-like cells, suggesting a possible association between microbial burden and alterations in Treg/Th17-related immune profiles. Particularly, AB treatment depletes *P. gingivalis* from both oral and blood reservoirs, represented by dendritic cells (DCs). Consequently, AB reduces the expansion of CD1C^+^CCR6^+^ inflammatory blood DCs, increases CCL20 levels in serum and decreases the frequency of IL-17A^+^FOXP3^+^ T cells and the boosting of IL-17^+^FOXP3^+^ T cells mediated by IL-1β and IL-6. In addition, AB treatment decreases IL-1R1 expression on Tregs, which promotes the expression of IL17^+^Foxp3^+^ T cells. The levels of Tregs, Th17, and IL17A^+^Foxp3^+^ T cells correlate with clinical parameters of periodontitis, namely PPD and CAL. These findings suggest that short-term antibiotic treatment may be associated with changes in systemic Treg/Th17-related immune parameters. However, given the small sample size, short follow-up, and exploratory nature of the study, they do not establish that Treg modulation was responsible for the observed clinical effects [118] (Table 4).

Overall, current interventional evidence indicates that conventional periodontal treatments may be associated with changes in Treg-related cytokines, cellular phenotypes, and Th17/Treg-related immune parameters. However, these interventions were not specifically designed to target Tregs, and the available evidence does not establish a causal role for Treg modulation in treatment response. Therefore, these findings should be considered preliminary and hypothesis-generating.

**Figure 3:**
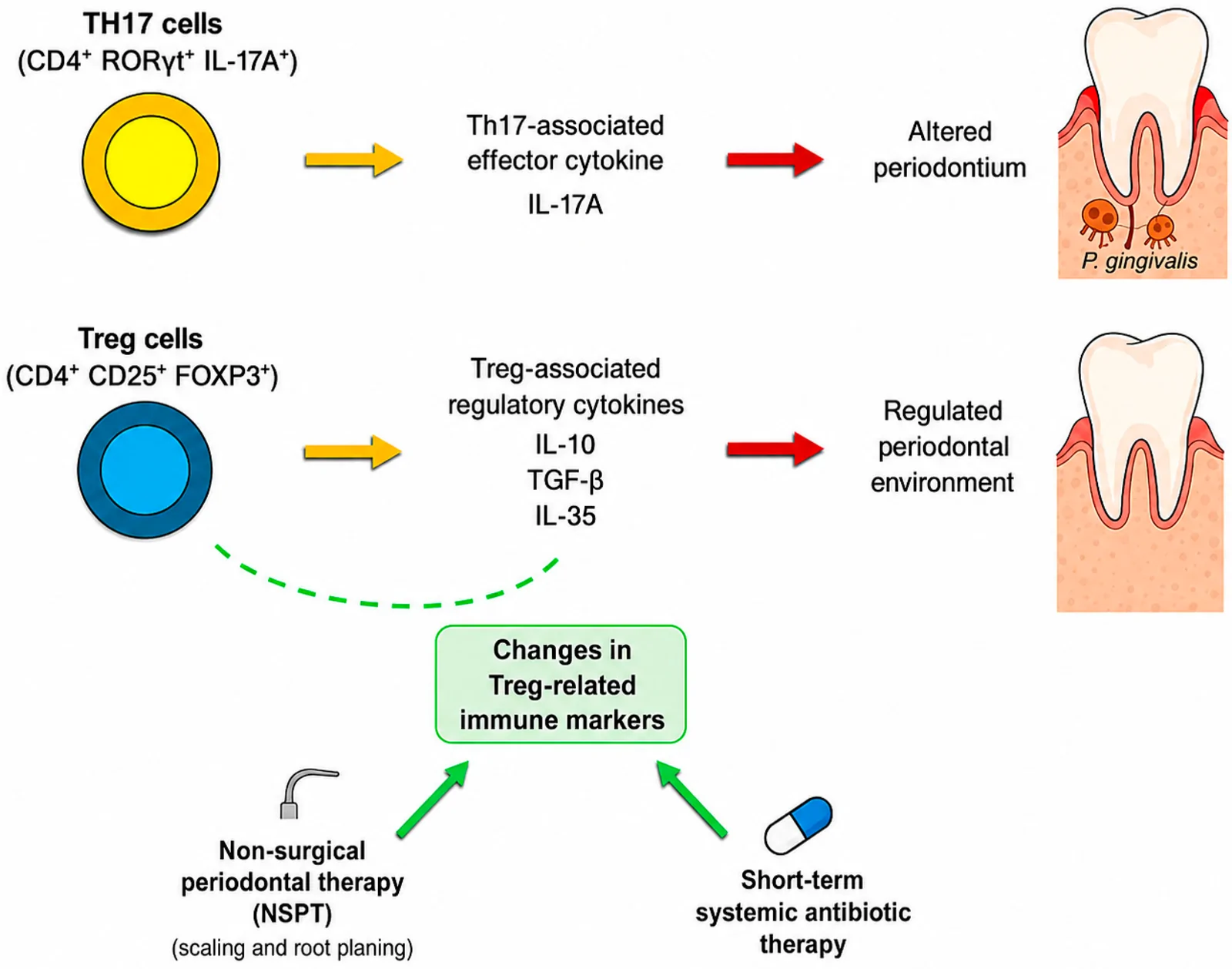
Proposed mechanistic model of Treg-related immune alterations in periodontitis. FOXP3: Forkhead box P3; IL-10: interleukin-10; IL-17A: interleukin-17A; IL-35: interleukin-35; RORγt: retinoic acid receptor-related orphan receptor gamma t; TGF-β: transforming growth factor beta; Th17: T helper 17 cells; Treg: regulatory T cell.

Figure 3 summarizes the role of Tregs in periodontitis, providing a schematic representation of altered Treg-related cytokine profiles and interventions to modulate Tregs and replace Th17/Treg imbalance.

CD4^+^ RORγt^+^ IL-17A^+^ Th17 cells are associated with pro-inflammatory signaling and periodontal tissue inflammation, whereas CD4^+^ CD25^+^ FOXP3^+^ Treg cells are associated with regulatory immune responses involving cytokines such as IL-10, TGF-β, and IL-35. The balance between Th17- and Treg-related responses may vary according to the clinical and biological context. Changes in Treg-related immune markers have been reported following nonsurgical periodontal therapy (NSPT) and short-term systemic antibiotic treatment; however, these interventions should not be considered established Treg-targeted therapies. From a pragmatic clinical perspective, the Treg-directed and immunomodulatory approaches discussed above remain investigational in periodontitis. Low-dose IL-2 and adoptive Treg-cell therapy require specialized clinical infrastructure and monitoring, with adoptive cell transfer representing a particularly resource-intensive strategy. HDAC inhibitors also remain experimental and may be limited by systemic and off-target effects. In contrast, vitamin D supplementation and probiotic or prebiotic approaches are more accessible and easier to integrate into clinical practice; however, current evidence does not establish that their potential periodontal benefits are specifically mediated through Treg modulation [119]. Therefore, none of these approaches can currently be regarded as an established Treg-targeted therapy for routine periodontal care, and their efficacy, safety, feasibility, and cost-effectiveness require evaluation in dedicated controlled clinical studies.

### 4.4. Limitations

Several limitations should be considered when interpreting the findings of this systematic review. The included studies showed clinical and methodological heterogeneity, particularly regarding study design, periodontal disease characteristics, biological specimens, Treg phenotyping, laboratory methods, and the presence of systemic comorbidities. Moreover, the included studies used heterogeneous definitions and diagnostic criteria for periodontitis, reflecting different classification systems and study periods. Some studies adopted terminology and diagnostic frameworks predating the 2017 AAP/EFP classification, which may limit the direct comparability of disease severity and immunological findings across studies. These differences should be considered when comparing results across studies and may partly explain the variability observed in Treg-related findings.

In addition, several included studies were observational or based on relatively limited sample sizes, and potentially relevant factors such as age, smoking, glycemic control, systemic therapies, and periodontal disease severity were not uniformly controlled. Therefore, causal interpretations should be made cautiously, particularly when evaluating the relationship between periodontitis, systemic conditions, and Treg-related immune alterations. Similarly, the available interventional studies mainly assessed treatment-associated changes in immune markers rather than therapies specifically targeting Tregs.

Finally, the review was limited to English-language studies published between 2015 and August 2025, in accordance with the predefined eligibility criteria.

The restriction to English-language publications may have introduced language bias and may have resulted in the exclusion of potentially relevant evidence published in other languages, thereby limiting the comprehensiveness of the review. Moreover, the heterogeneity of populations, biological specimens, immunological outcomes, and study designs did not allow a clinically meaningful meta-analysis; therefore, a narrative synthesis was considered the most appropriate approach. Despite these limitations, the available evidence provides a useful contemporary overview of Treg-related alterations in periodontitis and identifies relevant areas for future standardized prospective research.

## 5. Conclusions

The available evidence suggests that periodontitis is associated with alterations in the frequency and function of anti-inflammatory Treg cells and pro-inflammatory Th17 cells, together with changes in related cytokine profiles. Such immune dysregulation may contribute to local periodontal inflammation and tissue destruction and may also be involved in the systemic inflammatory burden associated with conditions such as diabetes mellitus, rheumatoid arthritis, atherosclerosis, and cancer. Available studies suggest that periodontal interventions, including non-surgical periodontal therapy and short-term antibiotic treatment, may influence Treg-related immune responses and cytokine profiles. In particular, modulation of Treg-associated cytokines such as IL-35 has been associated with improvements in clinical periodontal parameters and changes in inflammatory mediators. These findings provide a biological rationale for further investigation of immune-based approaches targeting Treg stability and function, rather than evidence supporting their current clinical use. Nevertheless, the overall strength of the available evidence remains limited. The included interventional evidence is based on small samples, short follow-up periods, heterogeneous methodologies, and studies with moderate-to-high risk of bias, precluding firm conclusions regarding long-term efficacy, safety, or clinical utility. Therefore, the observed changes in Treg populations and Treg-related cytokines should currently be interpreted primarily as mechanistic and hypothesis-generating findings. In particular, the available evidence does not support the routine clinical use of Treg-related cytokines as diagnostic or prognostic biomarkers, nor does it justify the introduction of Treg-targeted immunomodulatory strategies as adjunctive periodontal therapies. Larger, well-designed randomized controlled trials with standardized immunological and periodontal outcomes and adequate long-term follow-up are required before these approaches can be translated into clinical practice.

## Figures and Tables

**Table 1:** Summary of the databases, search concepts, limits, and records retrieved.

Database	Search Strategy	Boolean Combination	Limits	Records Retrieved
**PubMed/MEDLINE**	“periodontitis”; “periodontal disease”; “regulatory T cells”; “regulatory T lymphocytes”; “Treg”; “Tregs”	(“periodontitis” OR “periodontal disease”) AND (“regulatory T cells” OR “regulatory T lymphocytes” OR “Treg” OR “Tregs”)	English language; publication period: 1 January 2015–22 August 2025	11
**Scopus**	“periodontitis”; “periodontal disease”; “regulatory T cells”; “regulatory T lymphocytes”; “Treg”; “Tregs”	(“periodontitis” OR “periodontal disease”) AND (“regulatory T cells” OR “regulatory T lymphocytes” OR “Treg” OR “Tregs”)	English language; publication period: 1 January 2015–22 August 2025	169
**Web of Science Core Collection**	“periodontitis”; “periodontal disease”; “regulatory T cells”; “regulatory T lymphocytes”; “Treg”; “Tregs”	(“periodontitis” OR “periodontal disease”) AND (“regulatory T cells” OR “regulatory T lymphocytes” OR “Treg” OR “Tregs”)	English language; publication period: 1 January 2015–22 August 2025	178
**Cochrane Library**	“periodontitis”; “periodontal disease”; “regulatory T cells”; “regulatory T lymphocytes”; “Treg”; “Tregs”	(“periodontitis” OR “periodontal disease”) AND (“regulatory T cells” OR “regulatory T lymphocytes” OR “Treg” OR “Tregs”)	English language; publication period: 1 January 2015–22 August 2025	6
**Total**	—	—	—	**364**

The core search strategy was adapted to the syntax and indexing characteristics of each database, while maintaining the same search concepts and Boolean structure.

**Table 2:** Characteristics and main findings of studies evaluating Treg-related immune alterations in periodontitis.

Authors (Year)	Study Design	Population	Periodontal Diagnosis/ Diagnostic Criteria	Biological Specimen	Treg Definition/ Endpoint	Analytical Method	Major Findings	Risk of Bias
Bi J. (2024) [101]	Two-sample Mendelian randomization study	348,926 participants: 1740 P; 347,186 controls	Periodontitis GWAS phenotype (IEU OpenGWAS); individual clinical classification not applicable	GWAS summary-level genetic data	Treg-related immune-cell phenotypes, including CD39^+^/CD25^+^ T-cell traits	Two-sample MR; IVW, weighted median, MR-Egger; pleiotropy, heterogeneity and sensitivity analyses	MR supported potential causal associations between several immune-cell phenotypes, including Treg-related traits, and periodontitis	some concerns
Su X. (2020) [103]	Analytical cross-sectional study with in vitro experiments	93: 63 P (31 ≤ 40 years; 32 > 40 years); 30 HC	Chronic periodontitis; specific diagnostic criteria/classification not reported	Peripheral blood and serum; BMDC/T-cell co-culture	CD4^+^CD25^+^FOXP3^+^ Treg frequency; Th17 frequency; Th17/Treg ratio	Flow cytometry; ELISA; BMDC/T-cell co-culture ± LPS	↓ Treg and ↑ Th17/Treg ratio in P; imbalance greater in younger patients and associated with increased CD40 expression on DCs	some concerns
Cheng W.C. (2018) [104]	Analytical cross-sectional study	43: 13 HC; 15 chronic P; 15 aggressive P	Chronic/aggressive periodontitis; Page–Eke and Demmer–Papapanou case definitions; ≥2 non-adjacent interproximal sites with AL ≥ 6 mm at ≥2 teeth, with BOP	Peripheral blood: PBMCs and serum	CD4^+^CD45RO^+^CD25^+^CD127 ^ low Treg frequency; cytokine-producing T-cell subsets	8–10-color flow cytometry; intracellular cytokine staining; Luminex assay	No significant differences in circulating Treg frequency or major immune-cell subsets between P and HC; systemic cytokine profiles were also similar	some concerns
Danielsen A.K. (2024) [102]	Analytical cross-sectional study with ex vivo stimulation	85: 26 HC; 33 grade B P; 26 grade C P	Periodontitis initially classified according to 1999 criteria and reclassified according to 2017 AAP/EFP; grades B/C, predominantly stages III–IV	PBMCs	FOXP3^+^ Treg cells; IL-10-producing CD4^+^ T cells; T-cell cytokine responses	Ex vivo bacterial stimulation followed by flow cytometry	Grade B P showed ↓ FOXP3^+^ Tregs; grade-specific alterations in T- and NKT-cell cytokine responses were observed following bacterial stimulation	some concerns
Karthikeyan B. (2015) [109]	Analytical cross-sectional study	20: 10 advanced P; 10 HC	Advanced chronic periodontitis; PD ≥ 7 mm, CAL ≥ 5 mm, BOP, radiographic bone loss > 2/3 root length	Gingival tissue	FOXP3 and RORγt gene expression as Treg- and Th17- related transcriptional endpoints	RT-PCR	↓ FOXP3 expression and ↑ RORγt expression in P, indicating a shift toward a Th17-related transcriptional profile	High concerns
Jin Y. (2017) [105]	Analytical cross-sectional study	40: 20 P; 20 HC	Moderate-to-severe chronic periodontitis; PD/CAL and radiographic bone-loss criteria	PBMCs, periodontal tissue, GCF and serum	FOXP3 expression; IL-35 subunits IL-12p35 and EBI3; IL-35 protein	RT-qPCR; ELISA; clinical periodontal assessment	↑ FOXP3/IL-35-related mRNA expression and ↑ IL-35 protein in P; IL-35 levels inversely correlated with PD and CAL	some concerns
Jansson L. (2021) [106]	Cross-sectional study	163 patients with healthy and diseased tooth and implant sites	Localized/generalized periodontitis; PPD ≥ 4 mm, BOP and radiographic marginal bone loss ≥ 3 mm; Stage III–IV criteria	GCF and PICF	Treg-associated cytokines, including IL-35, IL-10 and IL-11; broader cytokine profile	Multiplex cytokine assay (Bio-Plex, Bio-Rad Laboratories); multilevel and regression analyses	Cytokine levels were frequently lower at healthy sites than at periodontitis/peri-implantitis sites; profiles at P and peri-implantitis sites were broadly similar	some concerns
Arul D. (2019) [100]	Analytical cross-sectional study	30: 10 HC; 10 gingivitis; 10 chronic P	Chronic periodontitis; CAL > 1–2 mm and radiographic bone loss	Gingival tissue	Naturally occurring CD4^+^CD25^+^FOXP3^+^ Treg proportion	Flow cytometry	↑ proportion of CD4^+^CD25^+^FOXP3^+^ nTregs in P compared with gingivitis and HC; no significant difference between gingivitis and HC	High concerns
Sabarish R. (2016) [99]	Pilot cross-sectional study	15: 8 chronic P; 7 HC	Generalized chronic periodontitis; AL ≥ 1 mm at >30% of sites and radiographic bone loss	Peripheral blood	CD45RB^+^CD4^+^CD25^+^FOXP3^+^ nTreg proportion	Flow cytometry	Higher circulating nTreg proportion in chronic P compared with HC	some concerns
Vitales-Noyola M. (2016) [110]	Observational cross-sectional study with functional assay	42 P patients (33 peripheral-blood and 9 gingival-tissue samples); 25 HC	Chronic periodontal disease; specific classification system not reported	Peripheral blood and gingival tissue	CD69^+^ Treg frequency and suppressive function	Six-color flow cytometry; immunofluorescence; immunohistochemistry; in vitro suppression assay	↑ CD69^+^ Tregs in blood and gingival tissue of P patients; frequency inversely correlated with CAL, while suppressive function was reduced	some concerns
Parachuru V.P.B. (2018) [107]	Analytical cross-sectional study	20: 10 chronic P; 10 HC/gingivitis	Chronic periodontitis; specific classification system not reported	Gingival tissue	FOXP3^+^CD4^+^ cells; Treg/Th17-related gene expression; TGF-β1 and IL-10	Immunohistochemistry; immunofluorescence; PCR array; ELISA	FoxP3^+^ cells predominated over IL-17^+^ cells; IL-17^+^ cells were mainly mast cells; ↑ TGF-β1 and IL-10 in P tissues	some concerns
Yuvashri P. (2024) [108]	Cross-sectional study	42: 14 HC; 14 non-smokers with P; 14 smokers with P	Periodontitis; PPD ≥ 4 mm and CAL ≥ 1 mm	Unstimulated saliva	Salivary IL-35 concentration as a Treg-associated cytokine endpoint	ELISA; clinical periodontal assessment	IL-35 was highest in non-smokers with P, lower in smokers with P, and lowest in HC; IL-35 correlated with periodontal clinical parameters	some concerns

BMDCs: Bone Marrow-Derived Dendritic Cells, BOP: Bleeding on Probing, CAL: Clinical Attachment Loss, *C. acnes*: *Cutibacterium acnes*, CD4^+^ T cells: Helper T Cells, CD8^+^ T cells: Cytotoxic T lymphocytes, CD: Cluster of Differentiation, CK: Cytokines, DCs: Dendritic Cells, ELISA: Enzyme-Linked Immunosorbent Assay, Foxp3: Forkhead box P3, F. nucleatum: Fusobacterium Nucleatum, GCF: Gingival Crevicular Fluid, GI: Gingival Index, GIN: Gingivitis Group, GWAS: Genome-Wide Association Study, HC: Healthy Controls, IFN-α2: Interferon-alpha 2, IFN-γ: Interferon Gamma, IL: Interleukin, IPA: Ingenuity Pathway Analysis, IVW: Inverse Variance Weighted, LPS: Lipopolysaccharide, MFI: Median Fluorescence Intensity, MR: Mendelian Randomization, nTregs: Naïve Regulatory T cells, NK: Natural Killer, OPLS: Orthogonal Projections to Latent Structure, pts.: Patients, *P. gingivalis*: *Porphyromonas gingivalis*, PBMC: Peripheral Blood Mononuclear Cells, PCA: Principal Component Analysis, PCR: Polymerase Chain Reaction, PD: Probing Depth, P: Periodontitis, PI: Plaque Index, PICF: Peri-Implant Crevicular Fluid, PLS-DA: Partial Least Squares–Discriminant Analysis, RORγt: Retinoic Acid Receptor-Related Orphan Receptor Gamma t, RT: Reverse Transcription, *S. epidermidis*: *Staphylococcus epidermidis*, SRP: Scaling and Root Planing, Tregs: Regulatory T cells, TNFSF13B: Tumor Necrosis Factor Superfamily Member 13B, TGF-β1: Transforming Growth Factor Beta 1, Th17: T Helper 17 Cells, Treg: Regulatory T Cells, TREM1: Triggering Receptor Expressed on Myeloid cells 1, TREM2: Triggering Receptor Expressed on Myeloid cells 2; ↑: increase; ↓: reduction; ^ low: low expression of the indicated cell-surface marker.

**Table 3:** Characteristics and main findings of studies evaluating Treg-related immune alterations in periodontitis associated with systemic diseases.

Authors (Year)	Study Design	Population	Systemic Condition	Periodontal Diagnosis	Biological Specimen	Treg Definition/ Endpoint	Analytical Method	Major Findings	Risk of Bias
Jiang L. (2022) [111]	Observational comparative multi-omics study	41 participants: 10 T2DM + P; 10 T2DM; 10 P; 11 HC	Type 2 diabetes mellitus (T2DM)	Periodontitis	Subgingival samples; peripheral blood	Treg/Th17 ratio; Treg frequency; PD-1/PD-L1 expression in CD4^+^ and CD8^+^ T cells	16S rRNA gene sequencing; UHPLC–MS metabolomics; flow cytometry	↓ Treg/Th17 ratio in T2DM and T2DM + P vs. P and HC; ↑ PD-1/PD-L1-related expression in T2DM groups; associated microbiome and metabolic alterations	some concerns
Li J. (2022) [112]	Cross-sectional observational study	107 participants: 43 T2DM + P; 23 T2DM; 20 P; 21 HC	Type 2 diabetes mellitus (T2DM)	Periodontitis	Peripheral blood	Th17 and Treg cell frequencies; Th17/Treg ratio; Th1/Th2 ratio	Flow cytometry; clinical periodontal and metabolic assessment	↑ Th1/Th2 and Th17/Treg ratios in T2DM; ↑ Th17 cells particularly in T2DM + P	some concerns
Fei C. (2025) [113]	Cross-sectional cohort study	62 patients with RA and P	Rheumatoid arthritis (RA)	Periodontitis stage II vs. stage III/IV	Saliva and serum	Treg-associated/immunoregulatory mediator profile; no direct Treg cell quantification	Human inflammation multiplex immunoassay panel; periodontal examination	Severe P (stage III/IV) showed ↑ inflammatory/immunoregulatory mediators; ↑ serum sCD30, IL-10, IL-19 and osteopontin, and ↑ salivary BAFF/TNFSF13B and IFN-α2	some concerns
Martínez-Martínez R.E. (2025) [114]	Cross-sectional observational study	80 participants: 20 HC; 20 P; 20 RA; 20 RA + P	Rheumatoid arthritis (RA)	Periodontitis	Peripheral blood/PBMCs; subgingival plaque	Th17 (CD4^+^IL-17A^+^) and Treg (CD4^+^FOXP3^+^) cell percentages	Flow cytometry; PCR quantification of *P. gingivalis*; clinical periodontal assessment	↑ Th17 cells and *P. gingivalis* burden in RA + P; ↑ PD and CAL; no significant differences in Treg percentages among groups	some concerns
Kajihara R. (2022) [116]	Cross-sectional observational study	61 participants: 14 HC; 17 P; 5 cancer; 25 cancer + P	Cancer	Periodontitis	Peripheral blood and serum	CD3^+^CD4^+^CD25^+^FOXP3^+^ Treg frequency; IL-6, VEGF, TGF-β1 and CCL22	Flow cytometry; serum immunoassays; periodontal assessment	↑ circulating Treg frequency in cancer + P vs. other groups; ↑ IL-6 in cancer + P; no significant differences in VEGF, TGF-β1 or CCL22	low concerns
Yang J. (2022) [115]	Retrospective observational study	128 participants: 32 Pg-AS; 32 AS; 32 Pg; 32 HC	Atherosclerosis (AS)	Periodontitis/*P. gingivalis*-associated periodontal status	Subgingival plaque; PBMCs; plasma	Treg (CD4^+^CD25^+^FOXP3^+^) and Th17 (CD4^+^IL-17A^+^) frequencies; Th17/Treg ratio; FOXP3, IL-10 and TGF-β1 expression; IDO activity	Flow cytometry; RT-PCR; ELISA; *P. gingivalis* detection; plasma kynurenine/tryptophan ratio	↑ Th17 and ↓ Treg frequencies with ↑ Th17/Treg ratio in Pg-AS; ↓ FOXP3, IL-10 and TGF-β1-related expression; ↓ IDO activity correlated with ↑ Th17/Treg ratio	some concerns

AS: atherosclerosis, BOP: bleeding on probing, C: cancer, CAL: clinical attachment loss, CCL22: C-C motif chemokine ligand 22, CK: cytokines, ELISA: enzyme-linked immunosorbent assay, FOXP3: forkhead box P3, GI: gingival index, HbA1c: glycated hemoglobin, HC: healthy control, IDO: indoleamine 2,3-dioxygenase, IFN: interferon, IgG: immunoglobulin G, IL: interleukin, P: periodontitis, PBMCs: peripheral blood mononuclear cells, PD: probing depth, PD-1: programmed cell death protein 1, PD-L1: programmed death-ligand 1, Pg: Porphyromonas gingivalis, PI: plaque index, pts: patients, RA: rheumatoid arthritis, RORγt: retinoic acid receptor–related orphan receptor gamma t, RT-PCR: reverse transcription polymerase chain reaction, sCD30: soluble cluster of differentiation 30, T2DM: type 2 diabetes mellitus, TGF-β1: transforming growth factor beta 1, Th17: T helper 17 cells, Treg: regulatory T cells, UHPLC–MS: ultra high-performance liquid chromatography–mass spectrometry, VEGF: vascular endothelial growth factor; ↑: increase of; ↓: reduction of.

**Table 4:** Studies evaluating changes in Treg-related immune markers following periodontal interventions.

Authors (Year)	Study Design	Population	Periodontal Diagnosis	Intervention/ Comparator/ Follow-Up	Biological Specimen	Treg Definition/ Endpoint	Analytical Method	Major Findings	Risk of Bias
Goswamy A. (2022) [117]	Prospective single-arm before–after interventional study	20 systemically healthy patients with generalized chronic P	Moderate-to-severe generalized chronic periodontitis	Full-mouth NSPT (SRP); comparison with pretreatment baseline; follow-up at 1, 2 and 3 weeks	GCF	IL-35 concentration as a Treg-associated cytokine endpoint; no direct Treg cell quantification	ELISA; PI, GI, PPD and CAL assessment	Clinical periodontal parameters improved after NSPT; GCF IL-35 progressively ↑ from baseline to week 3 and was negatively correlated with clinical periodontal parameters	hight concerns
Rajendran M. (2019) [118]	Pilot randomized clinical trial	18 patients with moderate-to-severe generalized P: AB group n = 8; non-AB group n = 10	Moderate-to-severe generalized periodontitis	Both groups received standard periodontal care; AB group additionally received oral metronidazole + amoxicillin for 7 days; follow-up 4–6 weeks	Peripheral blood; subgingival microbial samples	Treg and Th17 populations; IL-17A^+^FOXP3^+^ CD4^+^ T cells as Treg–Th17 plasticity endpoint; IL-1R1 expression on Tregs	Flow cytometry; dendritic-cell phenotyping; 16S rRNA/microbial analysis; cytokine-related analyses; clinical periodontal assessment	AB treatment was associated with ↓ *P. gingivalis*, ↓ circulating CD1C^+^CCR6^+^ inflammatory DCs, ↓ IL-1R1 expression and IL-17A^+^FOXP3^+^ T-cell plasticity, together with improvement in periodontal clinical measures	some concerns

AB: antibiotic therapy, CAL: clinical attachment loss, CCL20: C-C motif chemokine ligand 20, CD1C^+^CCR6^+^: inflammatory blood dendritic cells, CK: cytokines, DCs: dendritic cells, ELISA: enzyme-linked immunosorbent assay, FOXP3: forkhead box P3, GI: gingival index, IFN: interferon, IL: interleukin, P: periodontitis, PD: probing depth, PI: plaque index, RCT: randomized controlled trial, SRP: scaling and root planing; ↑: increase; ↓: reduction.

## Data Availability

No new data were created or analyzed in this study. Data sharing is not applicable to this article.

## Abbreviations

AB	Antibiotic therapy
BAFF	B-cell activating factor
CCL20	C-C motif chemokine ligand 20
CCL22	C-C motif chemokine ligand 22
CTLA-4	Cytotoxic T-lymphocyte antigen 4
CD4^+^	Cluster of differentiation 4
CD25^+^	Cluster of differentiation 25
DCs	Dendritic cells
FOXP3	Forkhead box P3
GCF	Gingival crevicular fluid
GI	Gingival index
HbA1c	Glycated hemoglobin
IDO	Indoleamine 2,3-dioxygenase
IL-1β	Interleukin-1 beta
IL-2	Interleukin-2
IL-4	Interleukin-4
IL-5	Interleukin-5
IL-6	Interleukin-6
IL-10	Interleukin-10
IL-13	Interleukin-13
IL-21	Interleukin-21
IL-17	Interleukin-17
IL-35	Interleukin-35
IFN-α2	Interferon-alpha 2
IFN-γ	Interferon-gamma
IPEX	Immune dysregulation, polyendocrinopathy, enteropathy, X-linked syndrome
MeSH	Medical subject headings
NSPT	Non-surgical periodontal treatment
PD-1	Programmed cell death protein 1
PD-L1	Programmed death protein-ligand 1
PI	Plaque index
PPD	Probing pocket depth
PRISMA	Preferred Reporting Items for Systematic Reviews and Meta-Analyses
PROSPERO	International Prospective Register of Systematic Reviews
RANKL	Receptor activator of nuclear factor kappa-B ligand
RCT	Randomized controlled trial
RORγt	Retinoic acid receptor-related orphan receptor gamma t.
ROBINS-I	Risk of bias in non-randomized studies
SRP	Scaling and root planing
STAT5	Signal transducer and activator of transcription 5
T2DM	Type 2 diabetes mellitus
TGF-β	Transforming growth factor beta
Th1	T helper 1 cells
Th17	T helper 17 cells
TNFSF13B	Tumor necrosis factor superfamily member 13B
Tregs/Treg	Regulatory T cells
